# Gamified, Automated Virtual Reality Exposure Therapy for Fear of Spiders: A Single-Subject Trial Under Simulated Real-World Conditions

**DOI:** 10.3389/fpsyt.2020.00116

**Published:** 2020-03-03

**Authors:** Philip Lindner, Alexander Miloff, Camilla Bergman, Gerhard Andersson, William Hamilton, Per Carlbring

**Affiliations:** ^1^ Department of Psychology, Stockholm University, Stockholm, Sweden; ^2^ Centre for Psychiatry Research, Department of Clinical Neuroscience, Karolinska Institutet & Stockholm Health Care Services, Stockholm County Council, Stockholm, Sweden; ^3^ Department of Psychology, Uppsala University, Uppsala, Sweden; ^4^ Department of Behavioral Sciences and Learning, Linköping University, Linköping, Sweden; ^5^ Mimerse AB, Stockholm, Sweden

**Keywords:** virtual reality, gamification, specific phobia, exposure therapy, self-help

## Abstract

**Background:**

Virtual Reality exposure therapy (VRET) is an evidence-based treatment of phobias and recent research suggests that this applies also to self-contained, automated interventions requiring no therapist guidance. With the advent and growing adoption of consumer VR technology, automated VR intervention have the potential to close the considerable treatment gap for specific phobias through dissemination as consumer applications, self-help at clinics, or as blended treatment. There is however a lack of translational effectiveness studies on VRET treatment effects under real-world conditions.

**Methods:**

We conducted a single-arm (n = 25), single-subject study of automated, gamified VRET for fear of spiders, under simulated real-world conditions. After setup and reading instructions, participants completed the automated, single-session treatment by themselves. Self-rated fear of spiders and quality of life served as outcome measures, measured twice before, and one and two weeks after treatment, and at a six-month follow-up. Session characteristics and user experience measures were collected at the end of the session.

**Results:**

Mixed-effects modeling revealed a significant and large (d = 1.26) effect of treatment-onset on phobia symptoms (p < .001), and a small (d = 0.49) effect on quality of life (p = .025). Results were maintained at a six-month follow-up (p > .053). The intervention was tolerable and practical. There were no significant correlations between any user experience measure and decrease in phobia symptoms (p > .209).

**Conclusions:**

An automated VRET intervention for fear of spiders showed equivalent effects on phobia symptoms under effectiveness conditions as previously reported under efficacy conditions. These results suggest that automated VRET applications are promising self-help treatments also when provided under real-world conditions.

**Pre-registration:**

Open Science Foundation, https://doi.org/10.17605/OSF.IO/78GUB.

## Introduction

Virtual Reality Exposure Therapy (VRET) works by presenting computer-generated, virtual equivalents of phobic stimuli in an immersive way, usually through use of a headset with stereoscopic displays covering both eyes, interactive to head movements, giving the illusion of being able to look around in the virtual world. VRET is structured in a manner similar as traditional exposure therapy (1): *via* graded, systematic exposure to feared stimuli under controlled conditions and without safety behaviors, the fear response gradually habituates and inhibitory learning occurs (2). Since the first clinical applications of VR technology in the early 2000's, meta-analyses have revealed that VRET is efficacious (3), and even on par with traditional exposure therapy (4). Importantly, results generalize to reduced fear of real-world stimuli (5), there are low rates of deterioration (6) and efficacy has been demonstrated also among adolescents (7). Clinicians appear to have a positive view of VR interventions (8–10), and some findings indicate that some patients may even prefer it to traditional exposure therapy when given a choice between the two, as well as lower rates of refusal (11).

Despite these promising results, as of yet, there have been few attempts to distribute VRET at a larger scale, or implement VRET in regular clinical settings (12). Arguably, the primary reason for this lies in the limitations of the past generation of VR hardware, which was both expensive and inaccessible, required a lot of auxiliary equipment and a high degree of technical skill to program and operate, and was heavy to wear for prolonged durations. With the release of consumer VR platforms, including mature digital ecosystems for development and dissemination of applications, these concerns in theory no longer apply, encouraging a new generation of VRET interventions that are scalable and can be integrated in regular care (13). A promising alternative approach enabled by this new technology is automated, self-guided VRET that provide a complete treatment package and includes no therapist guidance. Such applications, if released on ordinary digital marketplaces for VR applications, have the potential to disseminate effective, evidence-based treatment to unprecedented numbers. For example, a first-generation consumer targeted VR relaxation application, distributed on an ordinary digital marketplace, attracted more than 40,000 unique users over two years, even at a time when very few consumers had access to this technology (14).

To our knowledge, at least three automated VRET applications have been developed and evaluated in high-quality trials: two for fear of heights (15, 16), and one for fear of spiders (17, 18). In the latter study, we evaluated a novel, automated VRET application with gamified exposure tasks and level design, against gold-standard *in-vivo* exposure therapy using a randomized non-inferiority design, with behavioral avoidance of a real spider serving as the primary outcome measure. Results revealed that the VRET group showed large improvements on behavioral avoidance immediately after treatment (d = 1.49), albeit not as large as the *in-vivo* group (d = 2.39). However, the VRET group continued to improve during the follow-up period, and behavioral approach scores were non-inferior at both three- and twelve-month follow-ups (d = 2.01 and d = 2.27 for VRET and *in-vivo*, respectively, at twelve months) (18).

This randomized non-inferiority trial featured some design aspects that strengthened the validity of the treatment comparison, yet may at the same time have limited the generalizability of the VRET within-group effect to naturalistic conditions. First, only participants meeting DSM-5 diagnostic criteria for specific phobia were included, as assessed using a structured clinical interview during screening. While specific phobias are some of the most prevalent mental disorders, with a lifetime prevalence estimate of 7.4% (19), sub-syndromal fears are much more common (20). Thus, the vast majority of users are expected to have a sub-syndromal fear of spiders. Since baseline severity is typically positively associated with symptom decrease (larger room for improvement), treatment effects may be smaller with samples with sub-clinical fears.

Second, the primary outcome measure in the trial was a behavioral approach test (BAT) (21), which involved confrontation with a real spider regardless of treatment allocation. This was important in order to show that fear reduction from VR exposure translated to reduced fear of the in-vivo equivalent stimuli that participants would encounter in real life (5), and BAT outcomes are generally considered the gold-standard in phobia treatment evaluation. However, completing an *in-vivo* BAT prior to treatment may constitute a minor in-vivo exposure task in-itself, which would confound the estimate of the effect of VRET. Since the previous trial did not include a control group with the same BAT procedure but without treatment, or even additional administration of self-report measures after the BAT but before the treatment session, the degree of confounding is unknown. Clinical experience from running the trial did however suggest that some participants experienced the BAT as therapeutic, making it an important translational research question to examine treatment effectiveness without this possible confounder (which would not be present in real-life conditions), having already shown efficacy using a gold-standard BAT in the previous trial.

Third, in order to match the arms for non-specific therapist factors, and for ethical reasons (no other automated VRET study had been published at time of data collection), the VRET intervention was delivered with a “technician” (clinical psychology MSc student) in the same room at all times, who was instructed to provide emotional support if deemed necessary. This occurred in an estimated 20% of the sessions (18). The automated VRET application was designed for use at-home or alone in a clinical setting, meaning that no equivalent support would be available. Thus, it is unknown whether efficacy or even tolerability of the VRET intervention will generalize to effectiveness (real-world) conditions. Finally, by necessity, the original trial was randomized and it was not deemed feasible or ethically justified to blind participants to treatment options. The moderating effect of participant treatment preference was however not examined in the original trial. Past research indicates that many participants prefer VRET over *in-vivo* exposure when presented with both (11), and meta-analytic research has revealed a small moderating effect of participant treatment preference in general (22) (although most of this work has been done comparing psychotherapy to medication and thus may not generalize).

In sum, while specific design aspects of the original non-inferiority trial maximized the validity of the efficacy comparison between a gamified VRET intervention and gold-standard *in-vivo* exposure therapy, new translational research is needed in order to study the effectiveness of the this type of intervention when delivered under real-world conditions. For this purpose, we conducted a non-randomized, single-subject trial using the publicly available version of the VRET intervention, delivered with minimal human contact, and featuring only self-reported outcome measures.

## Methods

This study was approved by the Regional Ethical Review Board in Stockholm (2018/1640-32) and pre-registered at the Open Science Foundation (https://doi.org/10.17605/OSF.IO/78GUB). All included participants provided informed written consent at the start of the treatment session. Participants were not reimbursed, yet of n = 25 included participants, n = 12 participants were psychology students who received course credit for their participation.

### Participants and Procedure

The study was advertised on campus and public bulletin boards, social media, student mailing lists, and by contacting participants excluded from the previous VRET trial (18) for not meeting full specific phobia criteria. All advertisements directed potential participants to the study website, which featured full study information and the online screening battery, made available through a secure platform (23). Inclusion criteria included scoring 55 or above on the Fear of Spider Questionnaire (FSQ) (24) that served as primary outcome measure, being at least 18 years old, being able to understand both Swedish and English adequately, and available to travel to Stockholm University on one occasion. The minimal exclusion criteria included presenting a serious mental disorder requiring immediate care, any ongoing psychological treatment or non-stable psychoactive medication, or having vision or balance problems that would impair the VR experience.

A total of n = 42 completed screening, of which n = 26 were contacted by telephone and scheduled for a SCID diagnostic telephone interview (25) for specific phobia (adapted to DSM-5 criteria). These interviews were conducted by one of five last-year clinical psychologist students (quasi-random assignment), who all had received training in conducting structured diagnostic interviews, and who prior to study onset received additional training in specific phobia assessment, including standard-setting examples in order to maximize inter-rater reliability. The diagnostic interviews were carried out for descriptive purposes only, and the first n = 25 participants who completed the interview and scheduled a session were included. See **Table 1** for participant characteristics. Participants were mostly young adult women, less than a third of whom had any previous experience with VR. Included participants were assigned an additional online pre-treatment assessment to complete before the treatment session.

**Table 1:** Sample characteristics.

Variable	M (SD) or n (%)
Met full specific phobia criteria	20 (80%)
Age	25 (11.0)
Female	19 (76%)
Married or in relationship	20 (80%)
Highest completed education	
*High school (gymnasium)*	14 (56%)
*Post-gymnasial vocational training*	2 (8%)
*University*	9 (36%)
Primary occupation	
*Student*	16 (64%)
*Work*	8 (32%)
*Retired*	1 (4%)
Previous psychological treatment	6 (24%)
Previous psychoactive medication	2 (8%)
PHQ-9 score	4.64 (3.38)
GAD-7 score	4.72 (3.08)
Any gaming during average week	12 (48%)
Hours spent on gaming per week^*^	
*Computer*	3.33 (4.68)
*Console*	1.25 (2.73)
*Smartphone*	3.71 (3.00)
*VR*	0 (0)
*Other*	0.167 (0.577)
*Total*	8.46 (6.07)
Any previous experience with VR	8 (32%)

*Among those who reported any gaming.

Treatment was delivered in a single, three-hour session at Stockholm University. Participant were met by a session leader and taken to the experiment room, provided written informed consent, and were given standardized written instructions that covered overall procedure, practicalities, and how to deal with basic technical problem (such as needing to restart the application in case of freezing) and what to do if overwhelmed or experiencing cybersickness. Participants were also provided with a telephone number to reach the session leader in case of problems or early treatment completion. Before leaving the room, the session leader assisted the participant in putting on the headset and making adjustments. When 20 min of the session remained (or earlier in case of early treatment completion), the session leader returned and provided the participant with a link to an online questionnaire covering their experience and any issues that arose (see below). The therapist completed a similar questionnaire on how the session transpired (see below). Unlike in the previous trial (18), VRET participants were not given any instructions to progress with in-vivo exposure in the weeks that followed. One and two weeks after the session, participants completed online post-treatment assessments. An additional online follow-up assessment was administered six months after completing treatment.

Although the large effect size found in the previous trial on the same outcome measure (d = 1.33) meant that a sample size of n = 7 would be enough to detect (with 80% power and two-tailed p =.05) the same within-group effect with a paired t-test, we hypothesized a lower effect size in the current study for the reasons stated above. While n = 15 would be required to detect d = 0.80 with 80% power, we chose n = 25 to protect against sampling risk and to make correlational analyses possible (r > 0.5 with 80% power). This final sample size would give 99.99% power to detect d = 1.33 and 97% power to detect d = 0.80. Power calculations were performed using the GPower 3.1 software and based on a simplified yet near-equivalent analytic technique to the mixed effects modeling used in actual analysis (paired t-test on pre- and post-treatment scores, averaged across phase).

### Treatment

The consumer version of the original VRET application, developed by Mimerse and publicly available under the name *Itsy* at the Samsung and Oculus digital marketplaces, was used in treatment. Itsy was designed to be a complete, standalone intervention and includes both psychoeducation, a virtual therapist and spider expert, and a gamified level design with eight sequential main levels with increasingly realistic and frightening spiders (26), each with three gamified sublevels: a simply gaze-focusing task, one of eight simple games focused on helping a spider complete a task, and an interactive gaze-directed approach task. Users provide subjective units of distress ratings at the beginning and end of each task, and have the possibility to use a pause function and skip levels if needed. Once a level is completed, the next level becomes available. For more information, see the non-inferiority trial and study protocol (17, 18). The VR hardware used was the same as in our previous study: a Samsung Galaxy S6 and Gear VR headset (without a hand controller).

### Measures

All measures were completed online. Participants completed the Patient Health Questionnaire 9-item (27) and Generalized Anxiety Disorder 7-item (28) self-rating scales as part of the screening procedure, used only for descriptive purposes. The Swedish version of the Fear of Spiders Questionnaire (FSQ) (24) served as primary outcome measure and was administered at all measurement occasions. The FSQ features 18 items, with Likert response format score 1–7, meaning that the total score ranges 18–126. The Brunnsviken Brief Quality of life scale (BBQ) (29) served as the secondary outcome measure and was also administered at all measurement occasions. The BBQ measures satisfaction with, and importance of, six empirically derived life domains, and provides a weighted total score ranging 0–96, with higher scores corresponding to great subjective quality of life.

A series of measures of session characteristics (e.g. session duration, number of breaks, number of calls to session leader, etc.) and user experience were also collected through digital questionnaires completed at the end of the session by the session leader and participant (respectively). See **Table 3** for details. Cybersickness symptoms were measured using the Simulator Sickness Questionnaire (30). Sense of presence in the virtual environment was measured using the four-item Gatineau Presence Scale (31) with an adapted 0–10 item response format, the two subscales of which (presence and anti-presence) are presented separately since they correlated negatively and non-significantly (r = −.32, p =.14, n = 23).

### Analyses

Unconditional linear mixed effects models (including all available data), with random slopes and intercepts, were used to model outcomes as a function of time (two plus two measures), coded as either zero (prior to treatment phase) or one (after treatment phase). To assess the stability of scores within each phase before running the mixed models, paired t-tests were conducted; none were significant (all p > .15), suggesting stabile phases and appropriateness of a phase-based time variable in the mixed models. Clinically significant change was defined as a change exceeding two standard deviations (32). To test for moderating effects, the individual averaged pre-post score difference on the FSQ was correlated against the user experience measures that showed sufficient variation in scores. Finally, since there was no obvious candidate for a linear time coding for long-term effects that would give equidistance relative to the main effect of treatment, long-term effects were analyzed separately using generalized estimating equations (GEE) using all available post and follow-up data. This approach was preferred over mixed effects models since there were no repeated measures of the follow-up phase. Analyses were conducted in the R statistical environment using the *jmv*, *lme4* (33, 34) and *geeglm* (35) packages.

## Results

### Pre-Post Treatment Effects and Session Characteristics

**Figure 1:**
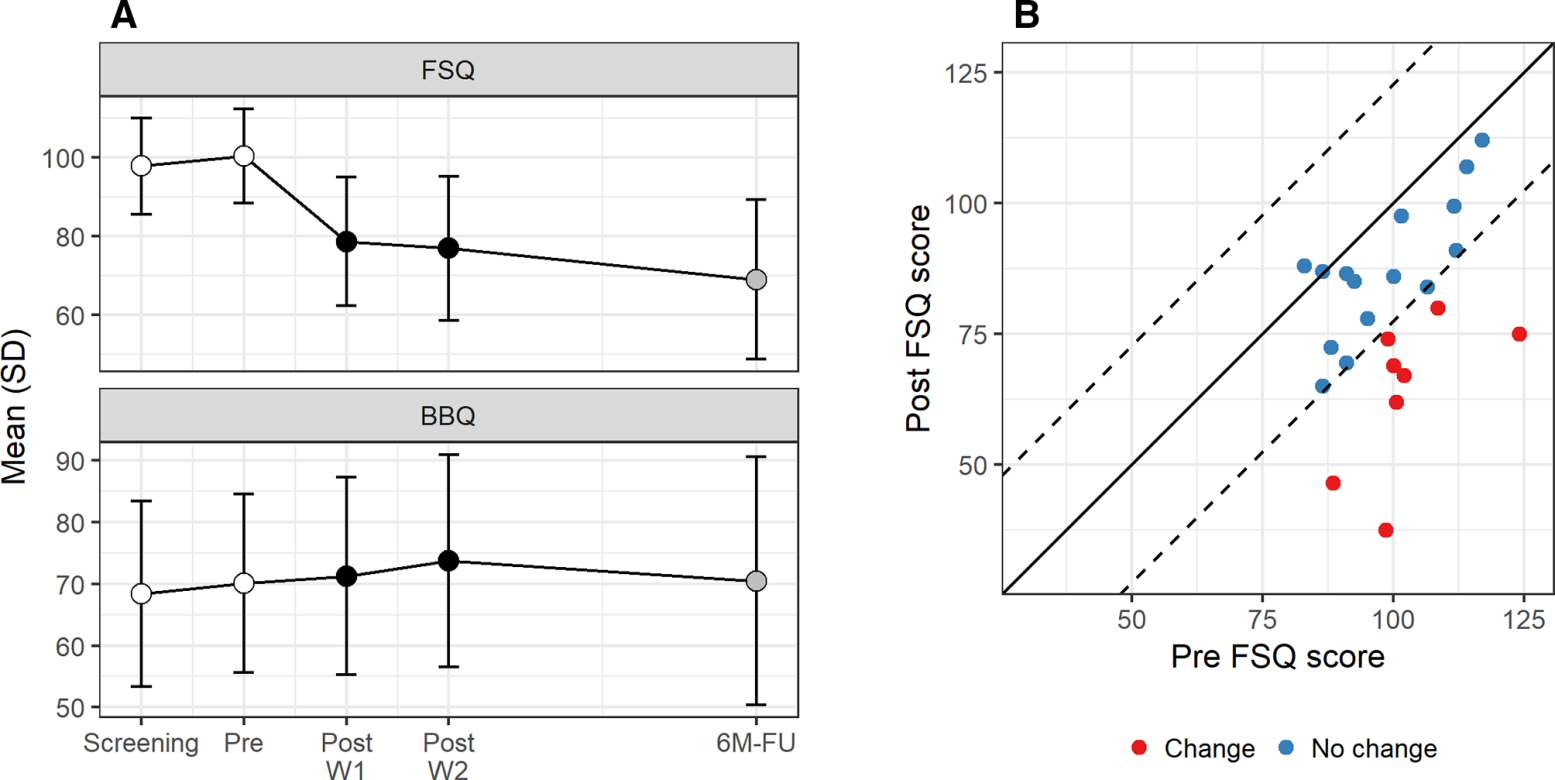
**(A)** Observed scores over time and **(B)** clinically significant change.

In total, n = 2 participants dropped out prior to treatment and missing data was at most n = 5 on any measurement point prior to follow-up. See **Table 2** and **Figure 1** for observed means and standard deviations. Mixed effects modeling revealed a significant effect of treatment-onset on FSQ scores (B = −20.57, SE = 3.46, p < .001, intercept-slope r = −0.18) and also on BBQ scores (B = 3.14, SE = 1.30, p =.025, intercept-slope r =.38), corresponding to observed within-group effect sizes of d = 1.26 (FSQ) and d = 0.49 (BBQ). Eight participants (35%) achieved clinically significant change.

**Table 2:** Observed means and standard deviations.

Time	Treatment-onset coding	Long-term effects coding	FSQ			BBQ	
			n	M	SD	M	SD
Screening	0	–	n = 25	97.80	12.26	68.36	15.01
Pre	0	–	n = 25	100.44	11.97	70.08	14.41
Post 1-week	1	0	n = 20	78.70	16.27	71.25	15.98
Post 2-week	1	0	n = 22	76.95	18.32	73.77	17.19
Six-month follow-up	–	1	n = 17	69.06	20.22	70.45	20.07

### Moderating Effects of User Experience

No significant correlations were found between FSQ score decrease and any measure of the user experience. See **Table 3** for details.

**Table 3:** User experience measures and session characteristics.

Session characteristics			
Variable	Rater	M (SD) or n (%)	
Completed session^a^	Session leader	23 (100%)	
Exposure duration (minutes)^b^	Session leader	97.22 (23.74)	
No calls to session leader	Session leader	20 (87%)	
Reasons for calls (multiple choice)	Session leader	–	
*Assistance with application required, but participant could have resolved issue themselves*		3 (12.5%)	
*Assistance with equipment required, but participant could have resolved issue themselves*		1 (4.2%)	
*Application malfunction (not resolvable by participant)*		0 (0%)	
*Equipment malfunction (not resolvable by participant)*		0 (0%)	
*Participant too distressed to continue and received basic emotional support*		0 (0%)	
*Participant too distress to continue and received support requiring clinical expertise*		0 (0%)	
*No call made*		20 (83.3%)	
No required visits by session leader (frequency)	Session leader	21 (91.3%)	
Use of in-game pause function	Participant	–	
*None*		10 (43.5%)	
*Yes: too distressing*		3 (13%)	
*Yes: other reasons*		8 (34.8%)	
*Yes: by accident*		2 (8.7%)	
Levels restarted or skipped (frequency)^c^	Participant	0.77 (1.31)	
Application restarts (frequency)	Participant	0.30 (0.56)	
All ten levels completed (frequency)^d^	Participant	22 (95.7%)	
In-session breaks (frequency)	Participant	1.52 (1.31)	
Total duration of breaks (minutes) ^e^	Participant	5.77 (5.53)	
**User Experience Measures**			
**Variable**	**Rater**	**M (SD) or n (%)**	**Correlation with FSQ score decrease**
Language comprehensibility	Participant	–	–
*No difficulties*		21 (91.3%)	
*Some difficulties*		2 (8.7%)	
*Great difficulties*		0 (0%)	
Visual acuity (theoretical range: 0–10)^f^	Participant	6.04 (1.74)	r =.201, p =.357
Gatineau Presence Scale: positive score (theoretical range: 0–20)	Participant	12.39 (4.62)	r =.272, p =.209
Gatineau Presence Scale: negative score (theoretical range: 0–20)	Participant	13.83 (3.33)	r = −.229, p =.292
SUDs maximum (0–100)^g^	Participant	69.09 (24.49)	r =.054, p =.805
SUDs final (0–100)^g^	Participant	48.57 (32.23)	r = −.035, p =.874
SUDs maximum minus final (habituation)	Participant	20.5 (21.6)	r =.11, p =.60
Cybersickness score (range: 0–48)	Participant	10.43 (6.32)	r =.084, p =.705

a Defined as either completing all levels or using all allocated time.

b Time from session leader leaving the room to either receiving a call from the participants (all levels finished) or ending the session when available time had elapsed.

c One outlier who reported 20,000 restarts removed.

d The remaining participant completed nine levels.

e One outlier who reported 90 minute pause removed.

f Verbal anchors “lousy” (0) and “perfect” (10).

g Participants asked to remember the subjective units of distress (SUD) reported in-app during application use, both the maximum and final value.

### Long-Term Effects

A total of n = 17 participants completed the six-month follow-up. GEE modeling revealed no significant score differences between post-treatment and six months later on the BBQ (B = −1.68, SE = 3.59, p = .64) and a trend towards further symptom decrease on the FSQ (B = −6.44, SE = 3.32, p = .053). See **Figure 1** and **Table 2** for observed means and standard deviations.

## Discussion

Virtual Reality exposure therapy has well-established efficacy, and several recent studies show that this applies also to automated interventions (15, 16, 18). Effectiveness studies examining the effects of automated VRET interventions under real-world conditions are thus the next research step along the translational pipeline before dissemination and implementation into clinical and non-clinical settings can be recommended. The current study was designed as a follow-up study to an earlier randomized non-inferiority trial comparing automated VRET for spider phobia to gold-standard in-vivo exposure therapy. Despite differences in inclusion criteria (specific phobia not required), study design (no randomization), treatment administration (no technician present during treatment) and measurements (only self-reports; no confounding of behavioral approach tests with real spiders), we found remarkably similar treatment effects as in the earlier trial: d = 1.26 in the current study, d = 1.33 in the earlier. It should however be noted that participant characteristics and baseline severity were similar across studies. For example, even though meeting diagnostic criteria for specific phobia was not required for inclusion in the current study, 80% did.

Importantly, the current study shows that consumer-targeted VRET applications can have comparable effects also under simulated real-world conditions. Specific phobias show one of the largest treatment gaps, with few sufferers seeking treatment. With continued growth in consumer VR adoption, consumer-target VRET applications, disseminated through ordinary digital marketplaces, have the potential to make a substantial public health impact. Pending mass-adoption, automated VR applications could be provided at clinics or pharmacies, without the need for a qualified therapist. Automated VRET interventions could also be used as homework exercises between traditional therapy sessions, akin to so called blended internet interventions (36). While the current study simulated at-clinic conditions to the extent possible, evaluating effectiveness of VRET applications when used at-home, outside a clinical trial setting, should be considered a research priority. Since the VR modality is well-suited not just for treatment delivery but also for measurement collection [both self-rating scales and virtual behavioral approach tests (37–39)], distributing VRET applications through digital marketplaces and collecting users' consent to share outcome data anonymously with researchers, would enable perfectly naturalistic studies on real-world data on an unprecedented scale. For comparison, even during the early years of consumer VR technology (when adoption rate was relatively low), one mental health application accrued over 40,000 users over two years of availability (14). A sample size of this magnitude would allow A/B testing (randomized allocation to different versions during actual use) of even minor changes to treatment design and delivery, providing valuable insights not just into the mechanisms of VRET but exposure therapy in general (12).

Overall, session characteristics and user experience measures suggest that the VRET interventions is tolerable and practical, although a lack of comparison group or validated norm data makes it difficult to draw firm conclusions. Interestingly, although designed to be played through in around two and half hours (corresponding to the three-hour in-vivo protocol included in the original non-inferiority trial), average usage time in the current study was approximately one and a half (in addition to setup, summing-up and questionnaire time). It should be noted that even in three-hour, in-vivo exposure protocols (21), only between two and two-and-a-half hours are spent on pure exposure (including short breaks as necessary). Unlike in the original non-inferiority trial, no personnel were present in the room while the participant played through the VRET serious game. As evident by the comparable effect sizes achieved across trials, having such personnel present does not appear to have therapeutic benefits, and likely also decreased playthrough time by making outside-VR social interactions impossible. The increased time efficiency is promising for future iterations and dissemination efforts, yet more research is required to capture more specific associations between how users engage with VRET serious games, and outcomes, beyond simple elapsed time.

Somewhat surprisingly, the current study found no significant correlations between treatment outcomes and user experience measures. Of note, this may be a power issue, since an *a priori* power calculation revealed that a correlation coefficient r > .50 could be detected with 80% power and a sample size of n = 25. For example, previous meta-analytic research suggests an association between sense of presence and SUDs of r = .28 (40), which the current study was not powered to detect. The lack of association between fear reaction and in-session habituation is consistent with the extant literature on the mechanisms of exposure therapy in general, which suggest that factors such as inhibitory learning processes (not measured in the current study) are a stronger predictor of treatment outcomes than simple emotional evocation and processing (2, 41). Although the extant VR literature (not limited to VRET) is somewhat inconsistent, the balance of evidence suggests a negative association between cybersickness and sense of presence (42). Congruently, neither of these measures were associated with treatment outcomes in the current study. Participants used a modern VR headset, albeit one limited to three degrees of freedom (i.e. measuring head rotation only, not position), running a VR paradigm that did not involve any virtual first-person motion. The latter was an explicit design consideration (12) with both a therapeutic rationale (covert invasion of personal space by spiders being a common catastrophic belief) and a technical one in that sensory discrepancies between virtual and physical first-person motion is a prime driver of cybersickness (43) and should thus be avoided unless necessary. As expected, sample average cybersickness score was low, as was the standard deviation, indicating that cybersickness was not a significant issue in the current study and that a floor effect for this factor may explain the lack of association with treatment outcomes. More research is needed on what aspects of the user experience during VRET promotes symptom reduction both immediately *in-virtuo* and when transitioning to *in-vivo* exposure in daily life (44).

Interestingly, we found only a small, non-significant (p = .053) further decrease in phobia symptoms at the six-month follow-up. In the non-inferiority trial, the VRET group continued to improve as measured by both the behavioral approach test and the Spider Phobia Questionnaire (45), although not as measured by the FSQ. A marked difference between these two studies is that in non-inferiority trial, but not the current study, participants were given a standardized rationale on the importance of transitioning to in-vivo exposure and a worksheet for planning and evaluating in-vivo exposure exercises. The effect of adherence to this transition plan was however not empirically evaluated, and studying the impact of in-virtuo to *in-vivo* transitioning through randomized allocation and appropriate statistics (46) should be considered another research priority for the field.

## Strengths and Limitations

The current study complements the earlier randomized non-inferiority trial (18) in terms of strengths and limitations of study design. Limitations include a small sample size (although well-powered given the hypothesized and observed large treatment effects), no control group, only self-reported outcome measures, and no long-term follow-up. Strengths of the current study include examining intervention effects under simulated real-world conditions (no randomization, minimum human contact) in sample which also included participants with sub-syndromal fear of spiders, as well as repeated measures at each study phase, and a six-month follow-up. User experience measures were self-reported only, and correlation analyses with treatment improvement low-powered. With efficacy of the intervention already having been established, the current study in some aspects prioritized the translational aim of generalizability to real-world conditions, including relying only on self-reported outcome measures as not to risk confounding the effect estimate by including a pre-treatment BAT that may constitute a brief *in-vivo* exposure exercise in-itself, and would not be available to at-home users. A waiting-list control group would have given unbiased estimates of treatment effects even with an included BAT, but was not included in the current study. Including more measures per phase (e.g. to model a pre-treatment trajectory), and a multiple baseline design, would have allowed for stronger inferences about causality.

## Conclusions

Automated, gamified VRET for spider phobia, in the form of a consumer application, is tolerable and practical also when delivered under simulated real-world conditions, with effects (fear reduction) remarkably similar to those previously observed in a randomized trial under efficacy conditions. Our findings show that automated VRET is a promising treatment option for self-help use, delivered either at clinics (no qualified therapist required) or at-home as consumer-oriented applications.

## Data Availability Statement

The datasets generated for this study are available on request to the corresponding author.

## Ethics Statement

This study was reviewed and approved by Stockholm Regional Ethical Review Board. The patients/participants provided their written informed consent to participate in this study.

## Author Contributions

Designed the study: PL, AM, PC. Technical development of the intervention: WH. Carried out data collection: CB. Made important contributions to study design and interpretation of findings: CB, WH, GA. Drafted the manuscript: PL. Made significant contributions to manuscript: AM, CB, WH, GA, and PC.

## Conflict of Interest

Author WH is the founder and chief technology officer of Mimerse, a company specializing in developing VR interventions for mental health. Author PL has consulted for Mimerse but holds no financial stake in the company.

The remaining authors declare that the research was conducted in the absence of any commercial or financial relationships that could be construed as a potential conflict of interest.
